# The role of smart monitoring digital health care system based on smartphone application and personal health record platform for patients diagnosed with coronavirus disease 2019

**DOI:** 10.1186/s12879-021-05898-y

**Published:** 2021-02-27

**Authors:** Jong Hun Kim, Won Suk Choi, Joon Young Song, Young Kyung Yoon, Min Ja Kim, Jang Wook Sohn

**Affiliations:** 1 Division of Infectious Diseases, Department of Internal Medicine, Korea University College of Medicine, 73 Goryeodae-ro, Seongbuk-gu, Seoul, 02841 Republic of Korea; 2 Present Address: Division of Infectious Diseases, Department of Internal Medicine, CHA Bundang Medical Center, CHA University, Seongnam, Republic of Korea

**Keywords:** Novel coronavirus disease 2019 (COVID-19), Outbreak, Community treatment center, Digital health care monitoring system

## Abstract

**Background:**

The massive outbreak of the novel coronavirus disease 2019 (COVID-19) in Daegu city and Gyeongsangbuk-do, Republic of Korea (ROK), caused the exponential increase in new cases exceeding 5000 within 6 weeks. Therefore, the community treatment center (CTC) with a digital health care monitoring system based on the smartphone application and personal health record platform (PHR) was implemented. Thus, we report our experience in one of the CTCs to investigate the role of CTC and the feasibility of the digital health care monitoring system in the COVID-19 pandemic.

**Methods:**

The Gyeongbuk-Daegu 2 CTC was set up at the private residential facility. Admission criteria were 1) patients < 65 years with COVID-19, 2) patients without underlying medical comorbidities, and 3) COVID-19 disease severity of mild class. Admitted patients were placed under monitoring of vital signs and symptoms. Clinical information was collected using the smartphone application or telephone communication. Collected information was displayed on the PHR platform in a real-time fashion for close monitoring.

**Results:**

From Mar 3, 2020, to Mar 26, 2020, there was a total of 290 patients admitted to the facility. Males were 104 (35.9%). The median age was 37 years. The median time between the COVID-19 diagnosis and admission was 7 days. Five patients were identified and were transferred to the designed COVID-19 treatment hospital for their urgent medical needs. The smartphone application usage to report vital signs and symptoms was noted in 96% of the patients. There were no deaths of the patients.

**Conclusions:**

Our results suggest that implementation of the CTC using a commercial residence facility and digital health care technology may offer valuable solutions to the challenges posed by the COVID-19 outbreak.

## Background

The novel coronavirus disease 2019 (COVID-19) outbreak was initially identified in Wuhan, China, at the end of 2019, and it rapidly spread in other countries throughout the world, resulting in a global pandemic [1]. After the incubation period of COVID-19 which is generally within 14 days, symptomatic infection can occur [2, 3]. The hall mark of symptomatic COVID-19 infection is that there are various presenting symptoms including fever, myalgia, cough, sore throat, diarrhea, and loss of smell or taste, which can cause mild illness or may progress to critical illness [2, 3]. In the Republic of Korea (ROK), the first case of COVID-19 was reported in early January 2020, and the number of new cases was on the gradual increase until late February 2020 when large clusters of new cases were identified in Daegu city and the adjacent province of Gyeongsangbuk-do [4]. The massive outbreak of COVID-19 in Daegu city and province of Gyeongsangbuk-do caused the exponential increase in the new cases exceeding 5000 within 6 weeks [4]. As a result of the on-going outbreak and a sudden influx of patients diagnosed with COVID-19, there were shortages of hospital beds and other resources in the local hospitals in Daegu city and province of Gyeongsangbuk-do. Such strain on hospital resources led to unprecedented numbers of home isolation of COVID-19 patients. Also, there were deaths of COVID-19 patients under home isolation while awaiting hospital admission [5]. One of the possible causes of death was delayed recognition of clinical deterioration from lack of monitoring in home isolation. Therefore, to address the safety of home-isolated COVID-19 patients and the allocation of scarce hospital resources, the community treatment center (CTC) with a digital health care monitoring system was designed and implemented as a part of the triage and management efforts for the COVID-19 patients by the Government of the ROK [4]. The patients confirmed to have COVID-19 were categorized into four classes (mild, moderate, severe, extremely severe) according to the severity of clinical presentation [4]. The COVID-19 patients with moderate, severe, and extremely severe classes were immediately hospitalized for treatment, and the COVID-19 patients with the mild class were sent to the CTC for isolation under close monitoring [4]. As of Mar 18, 2020, there were 12 CTCs in active operation for the COVID-19 patients in the ROK [6]. This establishment of the CTC COVID-19 patients’ management system by the Government of the ROK created a unique opportunity to propose and assess the role of the CTC and the feasibility of the digital health care monitoring system in the setting of the COVID-19 pandemic. Thus, we report our experience in one of the 12 CTCs, the Gyeongbuk-Daegu 2 CTC, located in Gyeongju, Gyeongsangbuk-do, which was deemed the epicenter of the COVID-19 outbreak in the ROK.

## Methods

This cohort study was conducted on the patients with COVID-19 admitted to the Gyeongbuk-Daegu 2 CTC during March 2020. Healthcare providers at the Gyeongbuk-Daegu 2 CTC consisted of 7 physicians, 9 nurses, 9 nursing-assistants, and 1 radiologic technician. Physicians included 1 infectious diseases physician, 3 board-certified plastic surgeons, and 3 public health doctors from the Ministry of Health and Welfare. There was a total of 45 other supporting workers at the Gyeongbuk-Daegu 2 CTC. They included the staff of administration, disinfection, food delivery, and housekeeping. All healthcare providers and other supporting workers rotated every 2 weeks, and they underwent the COVID-19 viral test at the end of their two-week rotation. A nasopharyngeal swab real-time polymerase chain reaction (RT-PCR) test [Seegene Inc., Republic of Korea] was used to detect the COVID-19 virus [7]. The Gyeongbuk-Daegu 2 CTC was set up at Nonghyup Residence, owned by a private company. Guestrooms were remodeled to accommodate COVID-19 patients. Single rooms were provided for individual patients. Shared rooms were provided for a family with patients. Admission criteria were 1) patients < 65 years diagnosed with COVID-19, 2) patients without underlying medical comorbidities, and 3) COVID-19 disease severity of mild class defined as lack of high fever (refractory fever > 38 °C despite the use of antipyretics) or dyspnea symptoms without evidence of pneumonic infiltrates on chest X-ray or computed tomography [8]. The COVID-19 patients who had followings were excluded and sent to the government-designated COVID-19 treatment hospitals: 1) underlying medical comorbidities including diabetes mellitus, chronic kidney disease, chronic liver disease, chronic pulmonary disease, chronic cardiovascular disease, hematologic malignancy, solid organ cancer, solid organ transplantation, hematopoietic stem cell transplantation, receipt of immunosuppressant, HIV, morbid obesity, and pregnancy or 2) clinical presentation compatible as disease severity of moderate or severe class such as high fever, dyspnea symptoms or evidence of pneumonic infiltrates on chest X-ray or CT.

**Fig. 1:**
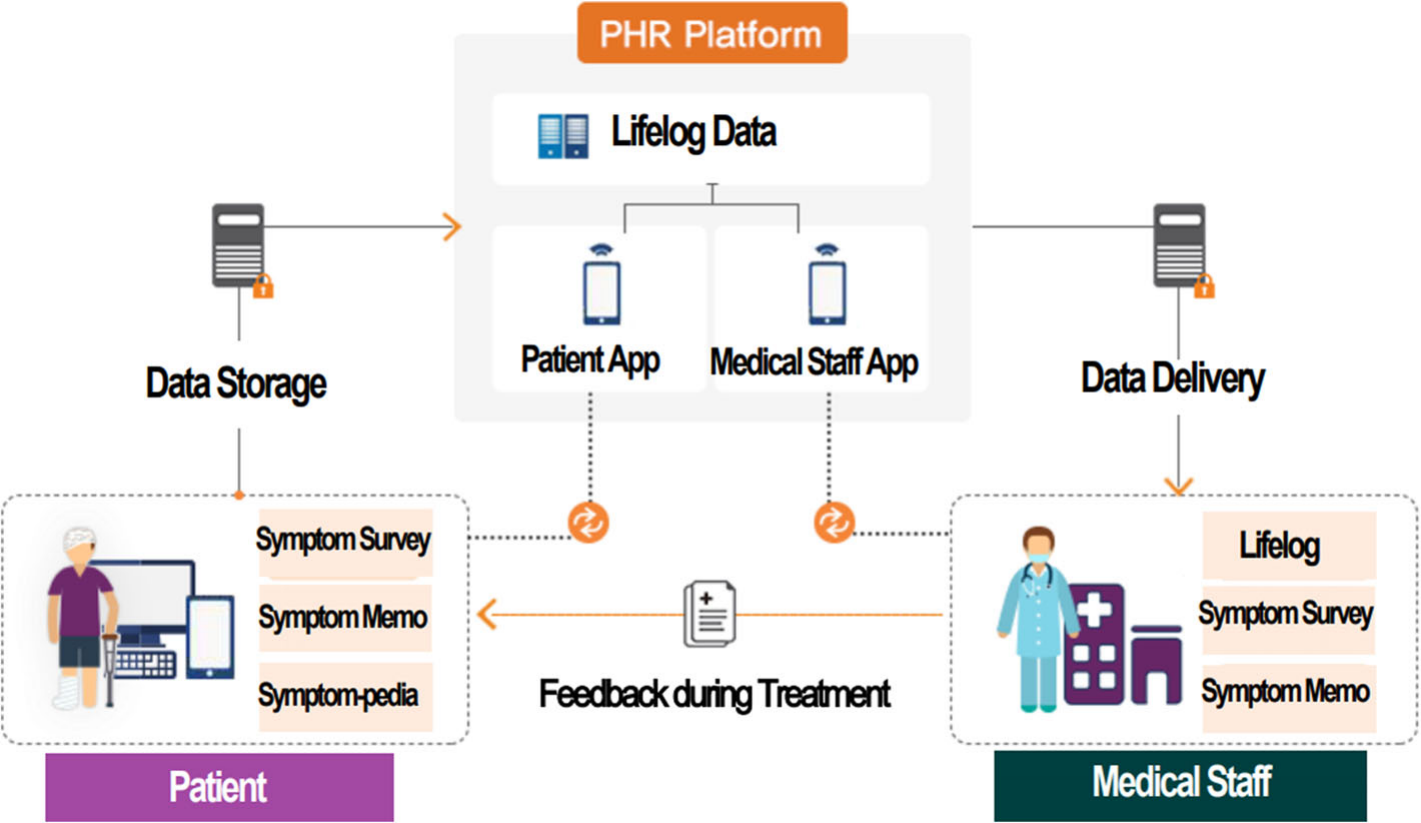
The flow of clinical data collection and communication using the smartphone application combined with personal health record (PHR) platform technology (Softnet, Seoul, Republic of Korea). Figure courtesy of Softnet Co., Ltd., Seoul, Republic of Korea, which is under copyright with the written permission given to use and adapt

Admitted COVID-19 patients at the Gyeongbuk-Daegu 2 CTC were placed under close monitoring of vital signs, including temperature, blood pressure, respiratory rate, and heart rate. Oxygen saturation was checked only in the case of the development of dyspnea symptoms. Each patient was provided with a personal thermometer, and the patient was instructed to check the temperature twice a day. Blood pressure and pulse rate were measured by a wireless blood pressure monitor device at the bedside. Oxygen saturation was measured by the nurse using a conventional pulse oximeter device. For the patients with the smartphone, a specialized smartphone application (inPHR®, Softnet Co., Ltd., Seoul, ROK) was provided and installed to their smartphones to report their measured body temperature and symptoms through the application. For the patients without the smartphone, telephone communication was used to report their body temperature. The patients’ other symptoms were reported via telephone communication or bedside rounding of healthcare providers. Clinical information was collected 1) automatically through the smartphone application and wireless blood pressure monitor devices or 2) manually by the health care providers after telephone communication or bedside rounding (Fig. 1). This collected clinical information was displayed on the dashboard using the integrated personal health record (PHR) platform (Softnet Co., Ltd., Seoul, ROK) for monitoring vital clinical information, including body temperature and symptoms. COVID-19 viral test was performed weekly though the nasopharyngeal and oropharyngeal swab. An RT-PCR test was used to detect the virus [7]. If the RT-PCR test was negative, then another RT-PCR test was performed 24 h later. If these two consecutive RT-PCR tests conducted at 24 h apart were both negative, then the patient was discharged from the facility. However, if there was abnormal body temperature or symptoms displayed in the dashboard, then it would give alerts to the health care providers for timely recognition and management of the patients. For the development of worsening respiratory symptoms or fever, chest radiograph and pulse oximetry were performed. If the patient was found to have pneumonic infiltrates or hypoxia or other urgent medical needs, then, the patient was transferred to the designated COVID-19 treatment hospital. If the patient did not meet the criteria for the transfer, then, the patient was provided with symptomatic treatment (e.g., antipyretics) since antiviral agents against COVID-19 such as remdesivir were not available.

Data were recorded using Microsoft Excel (Microsoft Corp, Redmond, WA, USA) and analyzed using SPSS software, version 18.0 for Windows (SPSS Inc., Chicago, IL, USA). Dichotomous variables were compared by the Pearson *χ*^2^ test or the Fisher exact test. For continuous variables, the Mann-Whitney test was used. A *P* < 0.05 was considered to be statistically significant.

## Results

From Mar 3, 2020, to Mar 26, 2020, there was a total of 290 COVID-19 patients admitted to the Gyeongbuk-Daegu 2 CTC. The majority of the patients were admitted on Mar 3, 2020 (235 patients, 81.0%), followed by the patients admitted on Mar 12, 2020 (19 patients, 6.6%) and Mar 14, 2020 (20 patients, 6.9%). Males were 104 (35.9%), and females were 186 (64.1%). The median age was 37 years (interquartile range (IQR) 26–50 years). The proportion of the patients < 19 years was 4.8% (14 patients). All of the patients had a confirmed diagnosis of COVID-19 infection. The median time between the COVID-19 diagnosis and admission to the Gyeongbuk-Daegu 2 CTC was 7 days (IQR 5–9 days). As of Mar 26, 2020, there were 5 patients who were transferred to the designated COVID-19 treatment hospitals. The reasons for hospital transfers included worsening underlying bipolar condition (1 patient), the rapid development of pneumonia (2 patients), an urgent need for prenatal care for pregnancy (1 patient), and need for pediatric care (1 patient, who was 2 years of age). More than half of the admitted patients were discharged in stable condition (187 patients, 64.5%) with the median duration of 10 days (IQR 8–18 days) at the facility. Between the patients who were remained at the facility (97 patients) and who were discharged (187 patients), there was no significant difference in terms of sex (males 32.0% vs. 38.0%, *P* = 0.317) and age (median 34 years vs. 37 years, *P* = 0.552). However, the duration of staying at the facility was longer for the patients who were remained at the facility than for the patients who were discharged (median 23 days vs. 10 days, *P* < 0.001). There were no deaths reported among the admitted patients at the facility. Also, there were no reported cases of COVID-19 infection transmission from the admitted patients to the healthcare workers and other supporting workers during the study period.

**Fig. 2:**
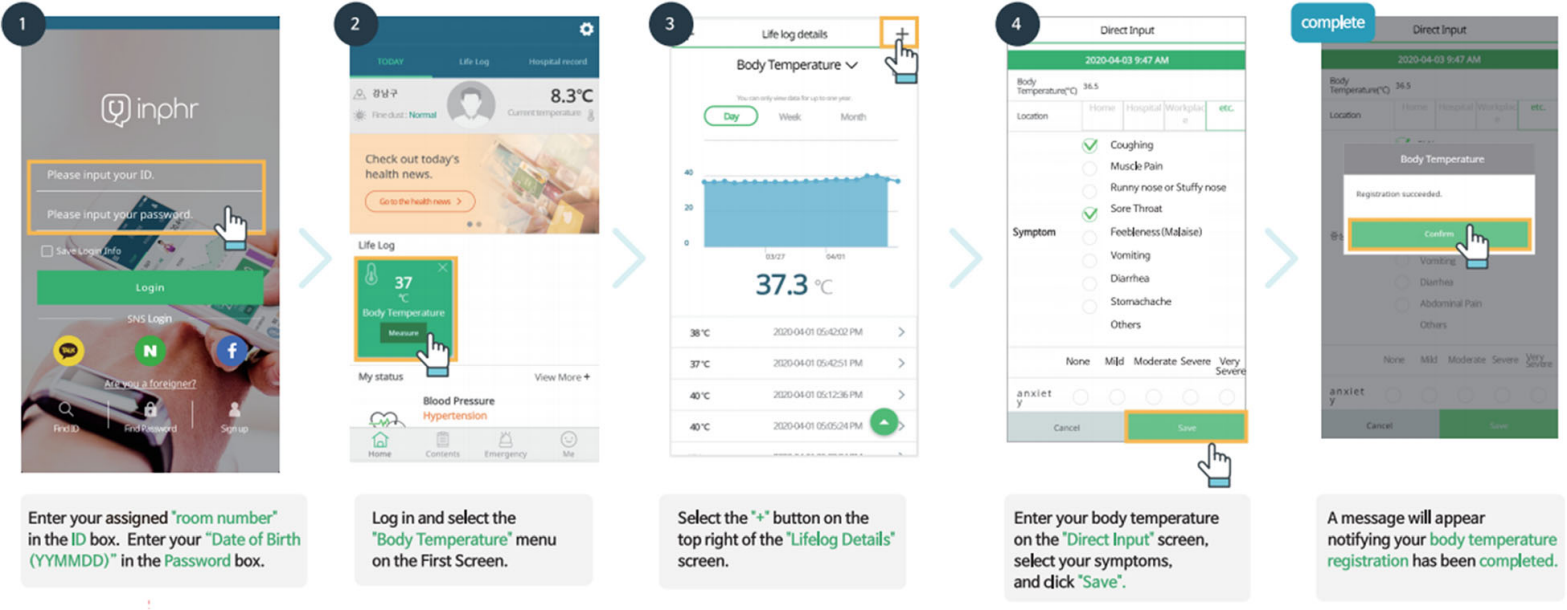
The smartphone application (Softnet, Seoul, Republic of Korea) used at the Gyeongbuk-Daegu 2 community treatment center (CTC). Figure courtesy of Softnet Co., Ltd., Seoul, Republic of Korea, which is under copyright with the written permission given to use and adapt

**Fig. 3:**
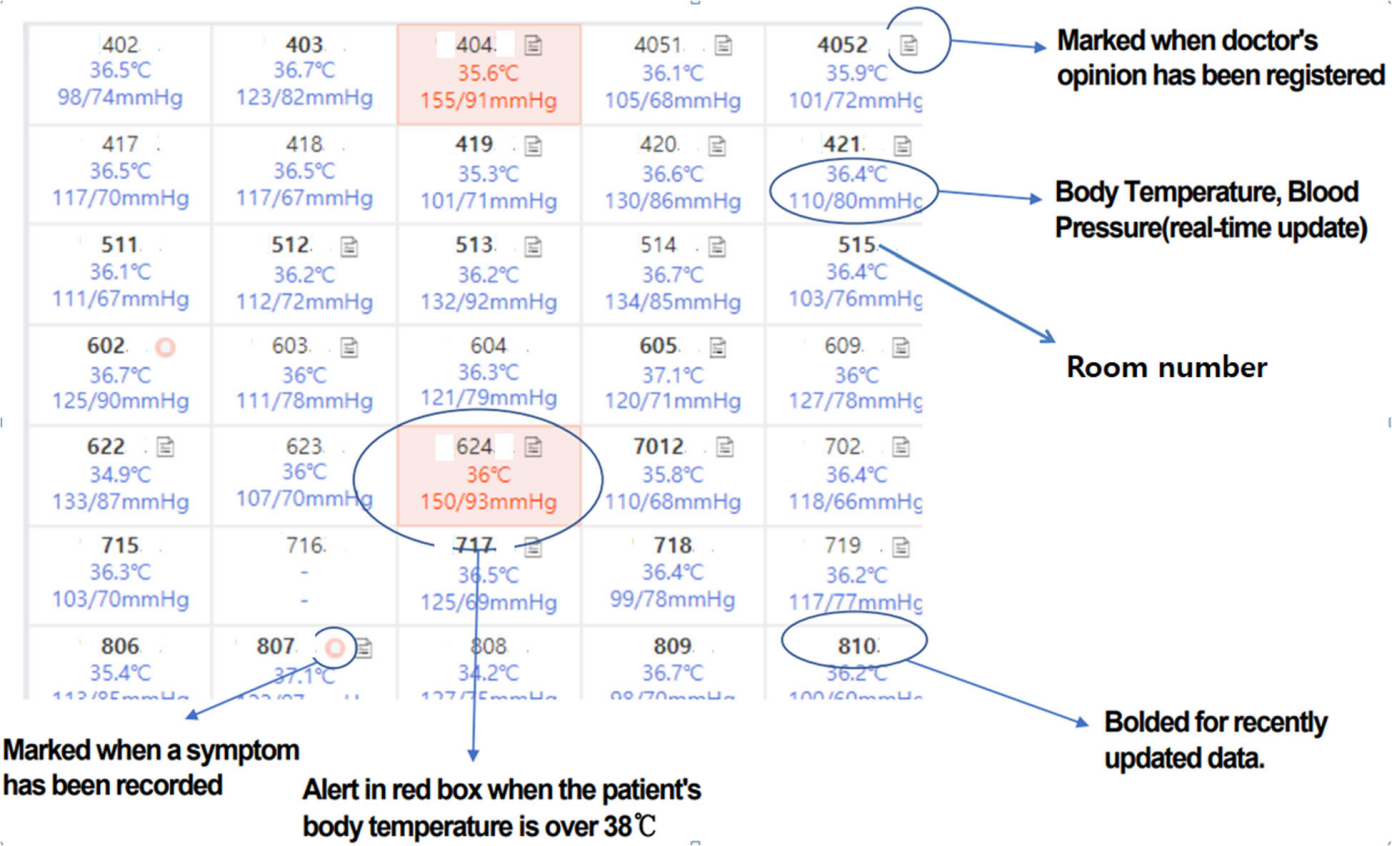
The overview of the dashboard of the personal health record (PHR) (Softnet, Seoul, Republic of Korea) used at the Gyeongbuk-Daegu 2 community treatment center (CTC). Figure courtesy of Softnet Co., Ltd., Seoul, Republic of Korea, which is under copyright with the written permission given to use and adapt

**Fig. 4:**
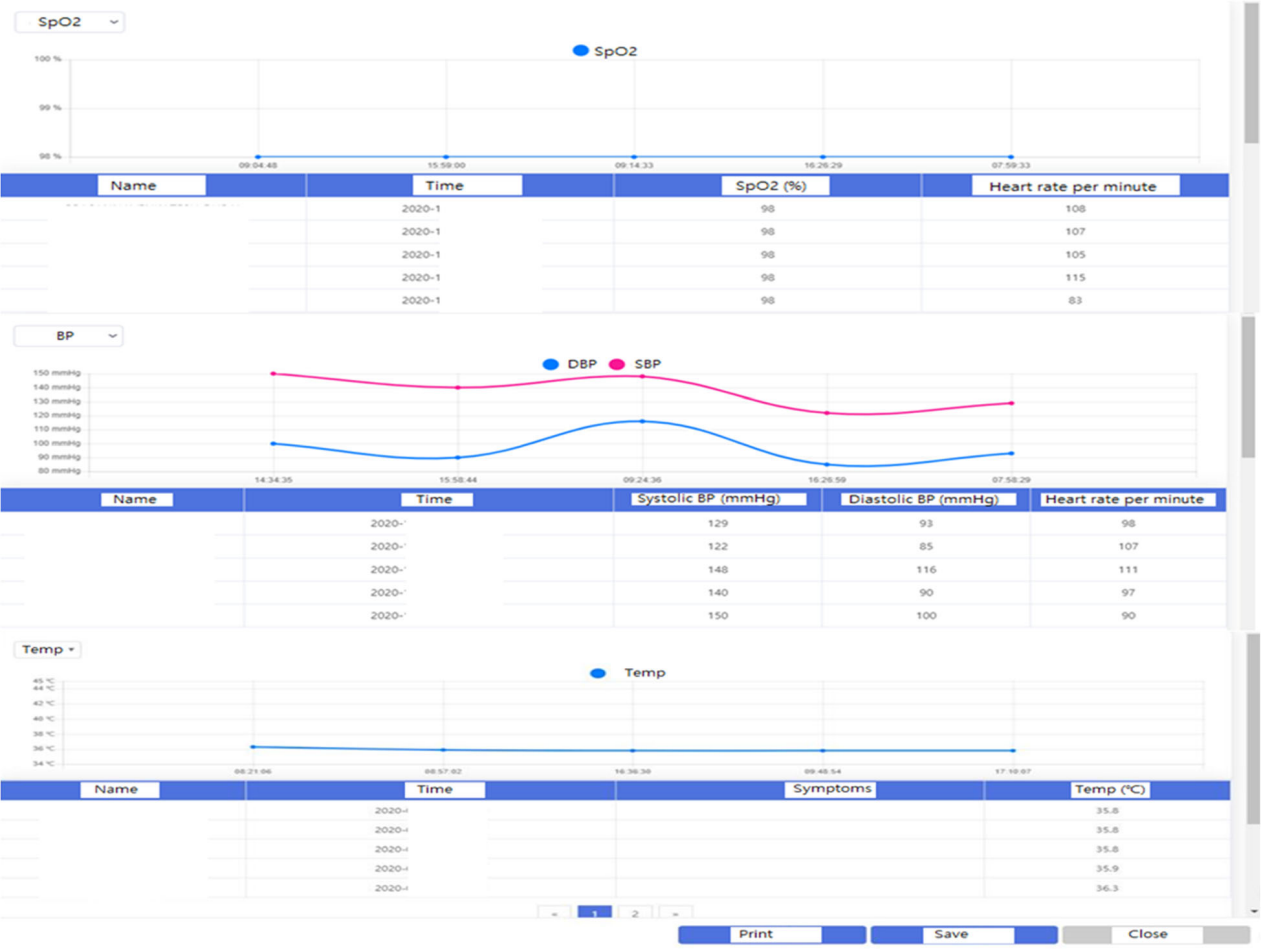
The details of the individual patient’s information displayed on the dashboard of the personal health record (PHR) (Softnet, Seoul, Republic of Korea) used at the Gyeongbuk-Daegu 2 community treatment center (CTC). Figure courtesy of Softnet Co., Ltd., Seoul, Republic of Korea, which is under copyright with the written permission given to use and adapt

Via the smartphone application, essential clinical information (temperature, blood pressure, and symptoms) recorded by the patients was converted to the lifelog common data in real-time. Reportable symptoms included coughing, muscle pain, runny nose, sore throat, malaise, vomiting, diarrhea, abdominal pain, and others (Fig. 2). The lifelog common data information was immediately available on the dashboard with the PHR platform for the healthcare providers after the patients completed their reporting of clinical information via the smartphone application. Among 290 patients, 279 patients (96.2%) used the smartphone application to report their symptoms. For the patients without the smartphone, clinical information was entered by the health care providers. The collected information was displayed on the dashboard in real-time as well. Therefore, real-time monitoring of the patients’ essential clinical information was available for the patients during the entire course of their stay at the Gyeongbuk-Daegu 2 CTC. As shown in Figs. 3 and 4, real-time updates of clinical symptoms and vital signs, comments from the health care providers, and alerts were displayed. This real-time monitoring process based on the PHR platform and the smartphone application was used in the identification of the patients at possible risks of clinical deterioration, including the patients who were transferred from the Gyeongbuk-Daegu 2 CTC.

## Discussion

Asymptomatic COVID-19 infections have been well documented, ranging from 50 to 90% of COVID-19 infections [9–11]. Furthermore, among cases of symptomatic COVID-19 infections, most symptomatic COVID-19 infections have been reported to be a mild class of infection (approximately 80%) [12]. Clinical guidance from the United States Centers for Disease Control and Prevention [13] states that COVID-19 patients with a mild infection may not require hospitalization. Thus, these patients may be placed on home isolation. However, COVID-19 patients with a mild infection under home isolation still require close monitoring of clinical symptoms as there is a possible risk of progression of COVID-19 infection to severe infection in the second week after the onset of symptoms [13, 14]. As previously reported [5], lack of monitoring in home isolation and delay in the hospital admission may contribute to morbidity and mortality of COVID-19 patients on home isolation when clinical deterioration occurs. Therefore, the CTC was implemented as a novel strategy to accomplish two objectives: 1) improvement of efficient allocation of medical resources to prevent the collapse of the health care system in the pandemic setting, 2) promoting the safety of COVID-19 patients with mild infection by providing quality medical monitoring and timely management services including a hospital transfer in case of clinical deterioration. Our experience at the Gyeongbuk-Daegu 2 CTC demonstrates that the CTC using a commercial residential facility can be utilized for accommodating COVID-19 patients with a mild infection, which would help reduce the burden of possible overcrowding and scarce resources of the designated COVID-19 treatment hospitals. In fact, the number of COVID-19 patients awaiting hospital admission at home was decreased from 3000 to less than 300 on Mar 18, 2020, after the implementation of several CTCs in Daegu city and province of Gyeongsangbuk-do [6]. This relief in the pandemic situation created by the implementation of the CTCs enabled the designated COVID-19 hospitals to concentrate their resources for the treatment of COVID-19 patients with a severe class of infection while preventing the shortage of hospital beds. Furthermore, the implementation of CTCs might help control further spread of COVID-19 infection in the community affected by the COVID-19 outbreak since isolation and distancing have been shown to be associated with the reduced transmission of COVID-19 infection [15].

Our experience at the Gyeongbuk-Daegu 2 CTC illustrates a new digital health care model equipped with the convenient smartphone application and PHR technology. Recently, smartphone applications have been used to assess various symptoms, including psychological symptoms and physical symptoms related to psychiatric disorders and medical illnesses [16–18]. Also, the PHR system has been used in the assessment and monitoring of several medical conditions in various settings [19, 20]. Therefore, the use of the smartphone application to report symptoms would be ideal for recording individual symptoms. Moreover, the clinical data collected via the smartphone application can be displayed in real-time fashion by the PHR technology, which would give valuable information for close monitoring. Furthermore, automated screening algorithms built into the PHR, such as the one used at the Gyeongbuk-Daegu 2 CTC, can be used to give alerts to the health care providers for timely management of the patients in cases of clinical deterioration. Successful identification and transfer of the patients in need of urgent care at the Gyeongbuk-Daegu 2 CTC suggest the practical utility and feasibility of the CTC model based on the new technology of smartphone application combined with the PHR in the setting of the COVID-19 outbreak. Another aspect of the CTC to consider is the issue of cost-effectiveness. Since the CTC can be set up at the private commercial residence in the community, it may avoid the excessive cost associated with building a new hospital facility. Furthermore, advanced technology of the smartphone application with the PHR at the CTC can offer the patients and health care providers to communicate effectively. This systemic advantage allows for the health care providers to monitor a larger number of the patients more effectively, which may lead to an improvement in care as well as reducing costs associated with monitoring in the COVID-19 outbreak setting, as suggested in the emergencies [21]. An additional advantage of the smart monitoring digital health care system may include that it can contribute to enhancing the safety of health care workers and other supporting workers. Substantial numbers of health care workers have been infected with COVID-19 infection from their exposure to the patients [22]. However, the smart monitoring digital health care system, such as the one used at the Gyeongbuk-Daegu 2 CTC, can minimize the physical contact with the patient during the process of monitoring. Thus, implementation of the smart monitoring digital health care system may reduce the risks of infection transmission associated with the physical contact. Our result of no reported cases of transmission of COVID-19 infection among the Gyeongbuk-Daegu 2 CTC personnel during the study period reaffirms an additional advantage of the smart monitoring digital health care system for the safety of health care workers and hospital personnel.

This study has some limitations, mainly due to a single-center study with a relatively small sample size and retrospective study design. Our CTC model with smart monitoring digital health care system has not been tested in the countries where there are higher COVID-19 disease burden rates than that of the ROK. However, we believe that our model can still be useful even in the setting of extremely large-scale COVID-19 outbreaks because it can contribute to the efficient allocation of medical resources and safety of both COVID-19 patients and health care providers. COVID-19 patients with underlying medical comorbidities were excluded from the study. Thus, unintended selection bias and confounding effects from unmeasured variables might have affected our results. Therefore, our results might not be generalizable to the clinical setting where there is a higher rate of underlying medical comorbidities or older population as COVID-19 patients with medical comorbidities or older age may have higher risks of progression of the infection [23, 24]. Moreover, we were not able to perform further comparison analysis between the patients who were remained at the facility and the patients who were discharged to determine the associated risk clinical factors. Such a study may be required in the future to identify the patients who are more likely to benefit from admission to the CTC during the outbreak of COVID-19. Nonetheless, we employed consistent definitions for data collection to minimize the potential bias of the study.

## Conclusions

In conclusion, the outbreak of COVID-19 infection in the community can lead to critical problems in health care. These problems include issues of allocation of scarce medical resources, risks of further spread of infection from the congregation of the patients, and risks of clinical deterioration on home isolation resulted in mortality from lack of monitoring. Our study results suggest that implementation of the CTC using commercial residential facility under smart monitoring digital health care system may offer valuable solutions to the challenges posed by the outbreak of COVID-19 infection.

## Data Availability

The datasets used and/or analyzed during the current study are available from the corresponding author on reasonable request.

## Abbreviations

COVID-19: Novel coronavirus disease 2019
ROK: Republic of Korea
CTC: Community treatment center
RT-PCR: Real-time polymerase chain reaction
PHR: Personal health record
